# Supplementary material for: Salicylic Acid Regulates Root Gravitropic Growth via Clathrin-Independent Endocytic Trafficking of PIN2 Auxin Transporter in Arabidopsis thaliana
Source: Int J Mol Sci. 2022 Aug 19;23(16):9379. doi: 10.3390/ijms23169379 (PMC9409447; doi:10.3390/ijms23169379)
Supplement: Supplementary file 1 [file ijms-23-09379-s001.zip › ijms-1856340-supplementary.pdf]

International Journal of
Molecular Sciences

MDPI

Supplementary materials:

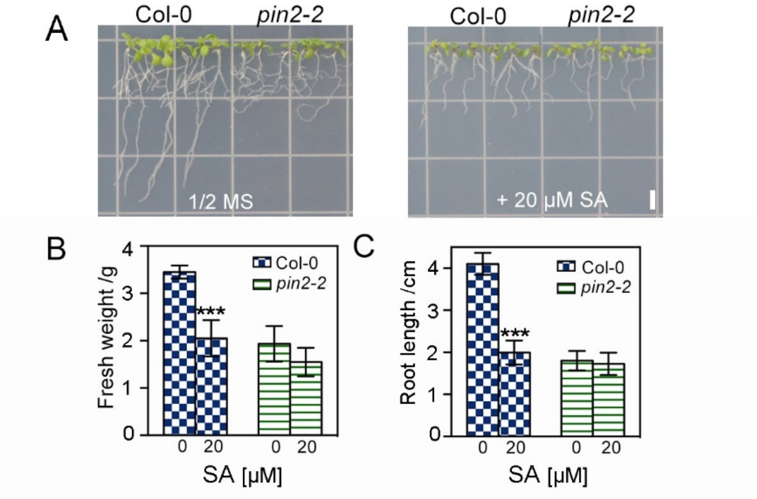

Figure S1. Effects of high-concentration SA on the root growth of Arabidopsis seedlings. Three-day-old wild type (Col-0) and *pin2-2* seedlings grown on ½MS medium were treated with 0 or 20 μM SA for 7 days. (A) Phenotypes of Col-0 and *pin2-2* seedlings. (B, C) Fresh weights and root length of Col-0 and *pin2-2* seedlings (n =6). Error bar = standard deviation (SD). P-values were determined with two-tailed Student's t-test assuming equal variances (***, P < 0.001).

Table S1. Primer sequences used for qRT-PCR analyses

Primers	5′-3′
PIN2-qRT-PCR-F	TTGCGGTTTTCGCGGTTCCT
PIN2-qRT-PCR-R	CGGCTAAACGCCTGCCAAAG
Actin-F	ACTCTCCCGCTATGTATGTCGCC
Actin-R	ATTTCCCGCTCTGCTGTTGTGGT
